# The role of PMA in enhancing the surface acidity and catalytic activity of a bimetallic Cr–Mg-MOF and its applications for synthesis of coumarin and dihydropyrimidinone derivatives†

**DOI:** 10.1039/d0ra03591b

**Published:** 2020-06-03

**Authors:** Reda S. Salama, Shawky M. Hassan, Awad I. Ahmed, W. S. Abo El-Yazeed, Mohammed A. Mannaa

**Affiliations:** Basic Science Department, Faculty of Engineering, Delta University for Science and Technology Gamasa Egypt reda.salama@deltauniv.edu.eg dr.reda.salama@gmail.com +201061391656; Chemistry Department, Faculty of Science, Mansoura University Mansoura Egypt; Chemistry Department, College of Sciences and Humanities in Al-Kharj, PrinceSattam Bin Abdulaziz University Al-Kharj 11942 Saudi Arabia; Chemistry Department, Faculty of Science, Amran University Yemen mnnaam@yahoo.com +967714152023

## Abstract

In the present study, a bimetallic Cr–Mg-MOF was successfully synthesized by the solvothermal method and then modified by loading different amounts of phosphomolybdic acid (PMA) using a simple wet impregnation technique. The morphological and structural properties of the prepared samples were investigated using X-ray diffraction, TEM, SEM, BET and FTIR spectroscopy. Importantly, Mg doping not only caused the Cr–Mg-MOF to have a higher surface area than MIL-101 (Cr) or MOF-74 (Mg), but the strategy of doping metal ions can be an effective way to improve the adsorption performance of MOFs. The surface acidity and the acid strength of the samples were determined using potentiometric titration and the FTIR of pyridine adsorption. The incorporation of PMA crystals gradually enhances both the surface acidity and the acid strength of the PMA/Cr–Mg-MOF catalysts up to 75 wt%. The catalytic performances of the prepared catalysts were tested in two acid-catalyzed organic transformations, namely, 7-hydroxy-4-methyl coumarin and 3,4-dihydropyrimidinone. In the two reactions, the catalytic activity attains the maximum value at 75 wt% PMA loading. The PMA catalysts supported on Cr–Mg-MOF are potentially promising heterogeneous catalysts for acid-catalyzed organic transformations in environmentally friendly processes, to replace the use of conventional homogeneous PMA catalysts.

## Abstract 2

PMA supported on Cr–Mg-MOF has been successfully synthesized. All catalysts exhibits distinct catalytic activity in the synthesis of 3,4-dihydropyrimidinones and 7-hydroxy-4-methyl coumarin and showed excellent % yield and recycling efficiency.

## Graphical abstract

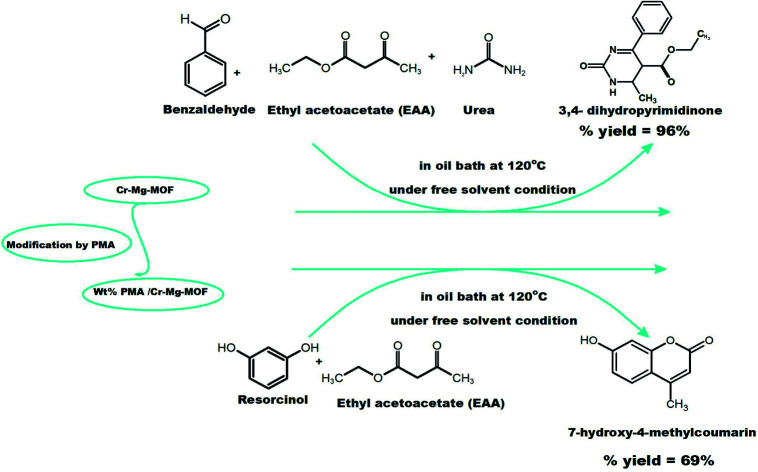

## 1. Introduction

One of the greatest goals in the catalysis research is producing highly active, feasible, economically and environmentally harmless heterogeneous catalysts due to the urgent need to remove the use of harmful materials.^1–3^ An example of this, liquid acid catalysts such as hydrofluoric acid, phosphoric acid and sulfuric acid were used in a number of chemical processes. It has become necessary to replace the liquid acid catalysts with solid acid catalysts from an environmental protection point of view,^4–6^ due to the corrosive properties of liquid acids. Heteropoly acids (HPAs) or polyoxometalates (POMs) are a large family of anionic metal–oxygen clusters of early transition metals and the most studied POMs are those showing the well-known Keggin structure [XM_12_O_40_]^*n*−^ (X = P, Si, Al, and M = W, Mo).^7–9^ They have attracted much attention as catalysts because of their redox properties and strong acidity, which can be adjusted by substituting the protons with metal cations and/or by altering the framework transition metal atoms or the heteroatom.^10,11^

12-Molybdophosphoric heteropoly acid (PMA), with the highest Brønsted acidity in the HPA series, displays as a potential heterogeneous catalyst for numerous acid catalyzed organic transformations, such as alkylation, hydration, hydrolysis, esterification, … *etc.*^10–14^ These solid acids has a very low specific surface area in the range of 1–5 m^2^ g^−1^ which making dispersion on a good support vital for ultimate application. So, immobilization of PMA onto porous supports becomes the commonly used method to heterogenize the HPA catalysts. Numerous porous supports, such as mesoporous molecular sieves,^14^ activated carbon^15^ and silica^16^ have been used to enhance the dispersion of heteropoly acid. One of the most important limitations of some of these supports are the absence of well-defined and the low loading of HPA crystals because of their tendency to aggregate over the surface of the support which leading to low catalytic performance.

Metal organic frameworks (MOFs) are crystalline porous hybrid substances built from metal or metal cluster coordinated to multi-dentate organic ligands called a linker leading to three-dimensional extended networks having channels and cavities.^17–20^ Their intrinsic high specific surface areas, permanent porosity, pore volume and tunable cavities make them applicable for numerous applications such as; catalysis,^19,21^ gas storage^22^ and separation.^23^ Due to the availability of different organic linkers and the coordination chemistry of the metal cations, permit metal organic frameworks to be an attractive platform to introduce various functional building blocks into one MOF framework for multifunctional applications. Mixed-metal approach over MOFs permits for combination of two or more different metals into a same framework to form mixed-metal MOFs,^24^ that offers MOFs with an additional degree of structural complexity and their properties are dependent on the incorporated metal atoms.

Coumarins are one of the most significant group of naturally occurring compounds mostly produced by different families in plants such as Rutaceae and Umbelliferae. In addition, coumarin derivatives show a wide range of applications, such as optical brighteners, food additives, perfumes, fragrances, agrochemicals, tunable dye lasers and cosmetics.^25–27^ They also have numerous convenient biological activities such as anticoagulant, insecticidal, anti-HIV therapy, antitumor properties.^28^ One of the most valuable methods for coumarin synthesis are Pechmann reaction in which very simple starting materials were used and produce excellent yields of numerous coumarin derivatives.^27,29^ Conventionally, coumarin derivatives can be synthesized by using different catalysts such as H_2_SO_4_, POCl_3_,^26^ W/ZrO_2_ solid acid catalyst,^30^ sulfamic acid supported on MIL-101 (ref. 31) and sulphate titania supported on MCM-41.^32^

Dihydropyrimidinones derivative establish a major family typically with pharmacological and therapeutic properties such as, antihypertensive medications, anti-carcinogenic, antimitotic, antiviral agents and especially, as modulators of calcium channels.^33–36^ The usage of catalysts in the condensation of Biginelli became essential to progress the time and the yields of reaction. We note in recent years, important efforts made with the purpose of discovery new procedures to yield 3,4-dihydropyrimidin-2(1*H*)-one derivatives with decent yields.

The present study aimed to prepare mixed component – metal organic framework by hydrothermal method and then modified by different weight contents of phosphomolybdic acid (PMA) by simple impregnation method. Numerous technique were used to elucidate the physiochemical properties of the modified catalysts such as XRD, FT-IR, SEM and TEM images while textural properties of these catalysts were measured by N_2_ adsorption at −196 °C. Pyridine adsorption and non-aqueous potentiometric titration with *n*-butylamine were also examined. The catalytic activity of PMA/Cr–Mg-MOF were then examined by using them as catalysts for the synthesis of 3,4-dihydropyrimidinones and 7-hydroxy-4-methyl coumarin.

## 2. Experimental

### 2.1 Materials

Chromium nitrate (Cr(NO_3_)_3_·9H_2_O, 98.5%) and magnesium nitrate (Mg(NO_3_)_2_·6H_2_O, 98.0%) were obtained from Sigma-Aldrich co., terephthalic acid (C_8_H_6_O_4_, 99+%) were purchased from Alfa Aesar. *N*,*N*-dimethylformamide (DMF ≥ 99.5%) and ethanol (≥99.5%) were commercially purchased from Alfa Aesar and used without further purification.

### 2.2 Synthesis of (Cr–Mg-MOF)

Typical synthetic process was carried out *via* solvothermal method as the following: a mixture of 2.4 g of chromium nitrate, 1 g of terephthalic acid and 1.54 g of magnesium nitrate were dissolved in 40 ml of DMF under stirring for 1 h and then the mixture was transferred to Teflon-lined steel autoclaves and heated up at 130 °C for 24 h. After cooling, green precipitate was formed. The precipitate was soaked in DMF for 1 day to remove unreacted terephthalic acid encapsulated inside the pores, then filtrated and washed by DMF and hot ethanol and finally dried at 120 °C under vacuum for 5 h.

### 2.3 Synthesis of PMA/Cr–Mg-MOF

Phosphomolybdic acid loaded on Cr–Mg-MOF was prepared by the direct impregnation method using different content of PMA (10, 25, 50, 75 and 90 wt%). In this method: 2.0 g of Cr–Mg-MOF was suspended in 50 ml of distilled water, sonicated for 0.5 h, and then stirred for 3 h. After that, definite amount of PMA was dissolved in 25 ml of H_2_O and then added the suspension under stirring for 8 h. After that, the suspension was left for 24 h under ambient temperature and then dried at 70 °C under vacuum. Finely, the powder was calcined at 150 °C for 3 h.

### 2.4 Catalyst characterization

X-ray powder diffraction pattern (XRD) of the as-synthesized catalysts were carried out using PW 150 (Philips) using Cu Kα radiation (*λ* = 1.540 Å) with a scan rate of 2° min^−1^. Fourier transform infrared (FT-IR) of all samples was recorded at room temperature using PerkinElmer system 2000 with a 4 cm^−1^ resolution and 128 scans in the middle IR region 400–4000 cm^−1^. The as-synthesized samples were degassed at 180 °C for 4 hours. The surface morphology of prepared samples was obtained by scanning electron microscopy (JEOL JSM-6510LV) and transmission electron microscopy (JEOL JEM-2100). N_2_ adsorption isotherms and BET surface area (*S*_BET_) of the as-synthesized catalysts were examined at −196 °C using BELSORP-mini II instrument. All the catalysts were evacuated at 150 °C for 2 h before measurements.

Total acidity and acid strength of the as-synthesized catalysts were examined by non-aqueous potentiometric titration. In which, 1 gram of activated samples was suspended in 10 ml acetonitrile for 2 h, then, titrated with 0.05 N *n*-butylamine in acetonitrile. Orion 420 digital model was used to measure electrode potential variation using a double junction electrode. FTIR spectra of chemisorbed pyridine was used to determine Brønsted and Lewis acid sites located on the surface of as-synthesized samples. About 0.1 gram of the samples were degassed at 200 °C for 3 h under high vacuum and then cooled to 30 °C. The dry pyridine was then flashed inside the vacuum system and the samples were maintained under these conditions for one month.^31^ Then, the excess pyridine was removed by evaporation at 70 °C after that, the samples were conducted using PerkinElmer system 2000 FT-IR spectrophotometer at 1400–1700 cm^−1^ by mixing 0.005 g of the sample with 0.1 g KBr in 30 mm diameter self-supporting discs.

### 2.5 Catalytic activities

#### 2.5.1 Synthesis of 7-hydroxy-4-methyl coumarin

In a general procedure, the desired coumarin was synthesized *via* Pechmann reaction in which a mixture of resorcinol (1 mmol), ethyl acetoacetate (2 mmol) and 0.05 g activated catalyst were transferred to 50 ml round flask then the flask was placed in an oil bath and refluxed at 120 °C under free solvent conditions (reaction monitored by TLC). The reaction mixture was filtered and pouring in 50 ml beaker containing crushed ice. The crude solid residue was recrystallized from ethanol to produce pure crystals of 7-hydroxy-4-methylcoumarin which characterized by FT-IR, ^1^H-NMR and melting point (186 °C). The effect of reaction parameters on conversion and product were studied. The effects of these parameters were studied at the same reactions conditions where the reactions examined over 0.05 g of 75 wt% PMA/Cr–Mg-MOF at 120 °C and the molar ratio of ethyl acetoacetate : resorcinol were 2 : 1.

#### 2.5.2 Synthesis of 3,4-dihydropyrimidin-2(1*H*)-ones

In a typical experiment, 3,4-dihydropyrimidin-2(1*H*)-ones were synthesized by mixing urea (1.5 mmol), ethyl acetoacetate (1 mmol) and benzaldehyde (1 mmol) in existence of 0.05 g of PMA/Cr–Mg–MOF catalyst without solvent. This reaction mixture was heated at 100 °C for the required time (monitored by TLC). After the reaction was completed, 10 ml of ethanol was added and the reaction was cooled to room temperature then transferred into a crushed ice. After that, the product was separated by simple filtration, washed by cold water and then recrystallized from ethanol to afford the pure product, which was identified, by melting point, FTIR spectra, ^1^H and ^13^C-NMR. The effect of reaction parameters on conversion and product were studied at the same reactions conditions. 75 wt% PMA/Cr–Mg-MOF was used and reaction mixture was heated at 100 °C and the molar ratio benzaldehyde : urea : ethyl acetoacetate were 1 : 1.5 : 1.

## 3. Results and discussion

### 3.1 X-ray diffraction pattern (XRD)

**Fig. 1:**
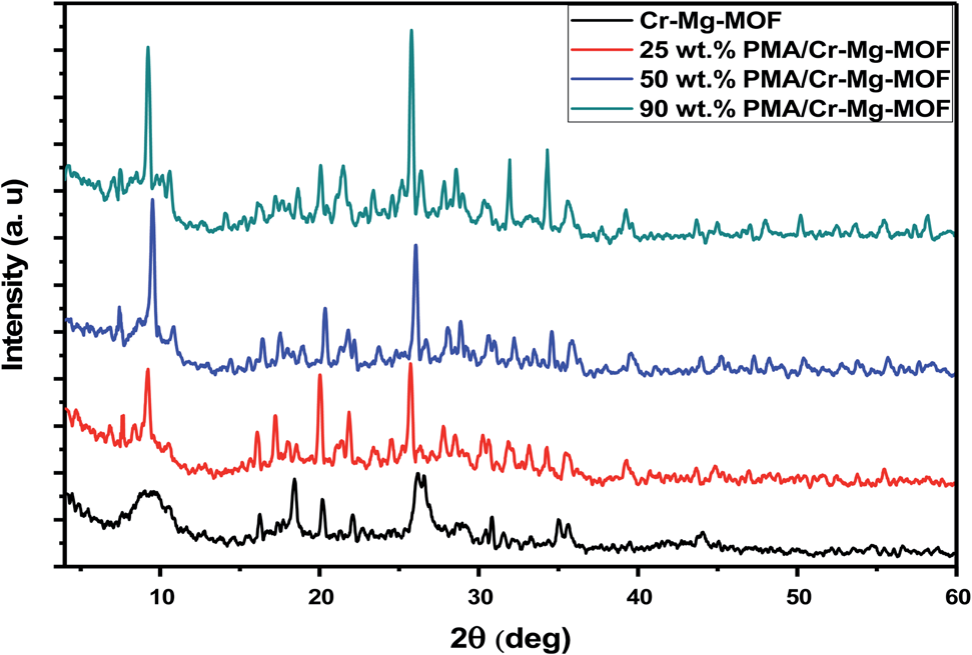
XRD pattern of pure and modified Cr–Mg-MOF by different wt% PMA.

XRD patterns of as synthesized Cr–Mg based MOFs catalysts were performed to confirm their structures. XRD patterns of mixed Cr–Mg – MOFs shows a series of diffraction peaks at 2*θ* equal 16.2, 18.3, 20.3, 22.1, 26.0 and 35.1° that agreed with XRD patterns early published.^31^ The diffraction patterns of the support clearly indicate that mixed MOFs were synthesized successfully. On the other hand, catalysts with 25, 50 and 90 wt% loading display highly crystalline nature with intense peak at 2*θ* equal 7.48, 9.61, 25.9, 32.07 and 34.89° that were assigned to the typical diffraction peaks of Keggin ion structure^37^ as displayed in Fig. 1. Also, the XRD patterns of pure Cr–Mg-MOF displayed a small shift in the peak positions after loading with PMA which may be related to the strong interaction of PMA with Cr–Mg-MOF.

### 3.2 Fourier transform infrared (FT-IR)

**Fig. 2:**
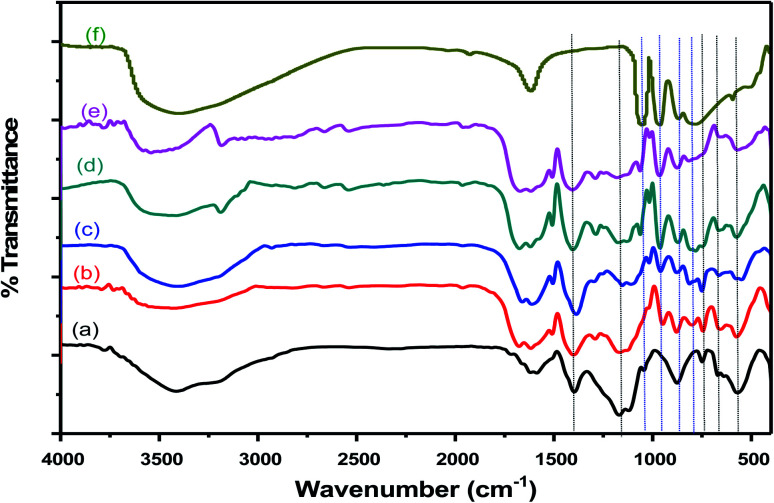
FTIR spectra of (a) mixed Cr–Mg-MOF; (b) 10 wt% PMA/Cr–Mg-MOF; (c) 50 wt% PMA/Cr–Mg-MOF; (d) 75 wt% PMA/Cr–Mg-MOF; (e) 90 wt% PMA/Cr–Mg-MOF and (f) pure phosphomolybdic acid.

FT-IR spectra of Cr–Mg-MOFs and PMA supported on Cr–Mg-MOFs catalysts were displayed in Fig. 2. The figure shows that mixed metal MOF was successfully synthesized according to the appeared peaks. The peak displayed at 1145 cm^−1^ attributed to C–O stretching vibration.^38^ Another peak at 1407 cm^−1^ attributed to symmetric stretching modes of carboxylic acid. Furthermore, *δ*(C–H) vibration of aromatic rings was existed through weak and narrow band at 748 cm^−1^.^39^ The peak at 651 cm^−1^ was related to outplane and inplane bending modes of carboxylic (COO^−1^) groups.^31^ The band with medium strength at 571 cm^−1^ was attributed to metal–oxygen (M–O) vibrations which approves the formation of the desired metal organic framework.^40^ Disappearance of strong absorption band at 1710 cm^−1^ shows that all carboxylic groups of the terephthalic ligand are deprotonated.^40,41^

On the other hand, FT-IR spectra of pristine 12-molybdophosphoric heteropoly acid (PMA) and PMA supported on Cr–Mg-MOFs were exhibited in Fig. 2F. The figure displays that the fingerprint region of PMo_12_ lies between 500 cm^−1^ to 2000 cm^−1^. Four characteristic peaks of PMA Keggin ion at 786, 870, 965, and 1059 cm^−1^ could be observed, which might be related to Mo–O–Mo, Mo–O–Mo, Mo=O and P–O respectively. Moreover, FT-IR spectra of PMA/Cr–Mg-MOF, Keggin structure of these substances were found to persist unchanged, despite minor intensify in some peak strengths, which approve that, PMA was successfully loaded on Cr–Mg-MOFs as XRD pattern also displayed.

### 3.3 Surface morphology

**Fig. 3:**
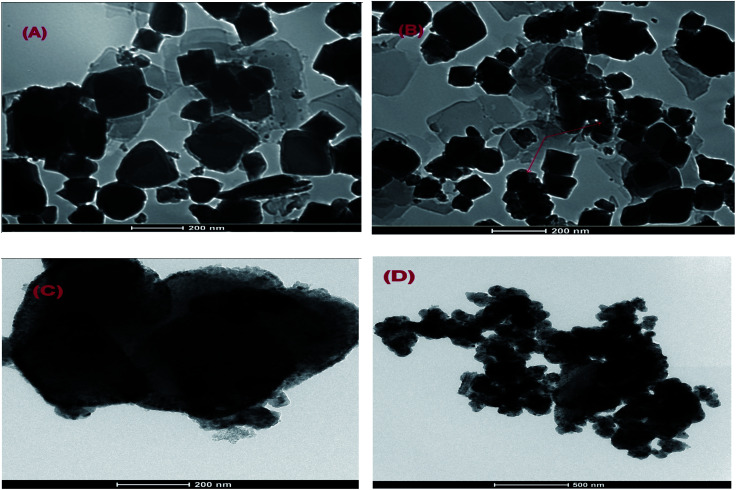
TEM images of (A) mixed Cr–Mg-MOF; (B) 25 wt% PMA/Cr–Mg-MOF; (C) 50 wt% PMA/Cr–Mg-MOF and (D) 90 wt% PMA/Cr–Mg-MOF.

To illustrate the surface morphology of Cr–Mg-MOFs and PMA supported on Cr–Mg-MOFs catalysts, transmission electron microscopy (TEM) and scanning electron microscopy (SEM) usually used. TEM images of unmodified Cr–Mg-MOFs displays distorted octahedral and spherical particles with size nearly 300–500 nm as shown in Fig. 3A. Morphology structure and uniform dispersion of PMA supported on Cr–Mg-MOFs catalysts were obviously revealed by TEM images which can be distinguished as dark dots. Fig. 3 displays TEM images of 25, 50 and 90 wt% PMA/Cr–Mg-MOF catalysts. Dark spots appear in the TEM images of Cr–Mg-MOF which related to some PMA within the pores and/or on the surface of supported catalyst. As well as, the number of dark spots increases within the increase in PMA contents.

**Fig. 4:**
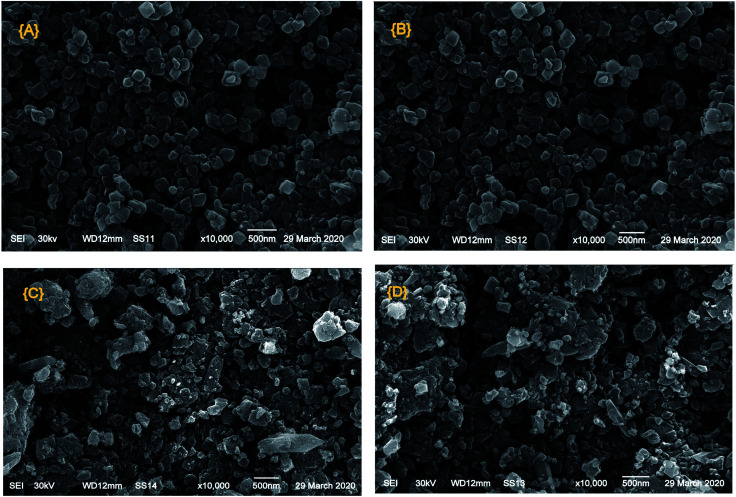
SEM images of (A) mixed Cr–Mg-MOF; (B) 25 wt% PMA/Cr–Mg-MOF; (C) 50 wt% PMA/Cr–Mg-MOF and (D) 90 wt% PMA/Cr–Mg-MOF.

Fig. 4 displays the SEM images of Cr–Mg-MOFs and PMA supported on Cr–Mg-MOFs catalysts. It was observed that the crystal particles of modified and unmodified catalysts still maintained octahedral and spherical structure as confirmed by TEM images. The SEM images show that 90 wt% PMA/Cr–Mg-MOF consists of larger particles than 25 wt% PMA/Cr–Mg-MOF which may lead to some diffusion limitation.

### 3.4 BET measurements

**Fig. 5:**
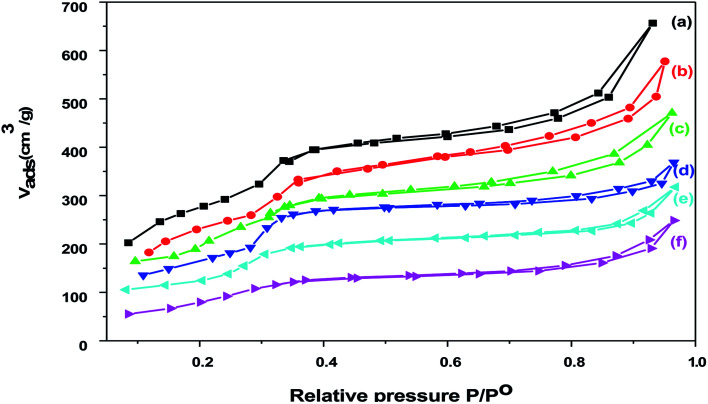
Adsorption–desorption isotherms of nitrogen at −196 °C on (a) mixed Cr–Mg-MOF; (b) 10 wt% PMA/Cr–Mg-MOF; (c) 25 wt% PMA/Cr–Mg-MOF; (d) 50 wt% PMA/Cr–Mg-MOF; (e) 75 wt% PMA/Cr–Mg-MOF; (f) 90 wt% PMA/Cr–Mg-MOF.

Nitrogen adsorption isotherms for Cr–Mg-MOFs and PMA supported on Cr–Mg-MOFs catalysts are shown in Fig. 5. The isotherms of Cr–Mg-MOFs catalyst displays type IV isotherm according to the IUPAC classification,^42,43^ which is characteristic of mesoporous materials with large pore volume (1.074 cm^3^ g^−1^) and high BET surface area (*S*_BET_ = 1524 m^2^ g^−1^). Little difference could be observed between the N_2_ sorption curves of Cr–Mg-MOFs and those of PMA supported Cr–Mg-MOFs. Suggesting that the introduction of PMA doesn't affect the mesoporous structure of Cr–Mg-MOFs. Table 1 shows the effect of PMA contents on the surface area of MOF sample. Addition of PMA to the support results in a decrease in the surface area. These reduction in surface area after loading may be due to the fact that the PMA is deposited inside the mesochannels and is well dispersed on the surface of Cr–Mg-MOFs support. The surface area per gram of the support indicated that the loading of PMA gradually decreased the surface area as displayed in Table 1.

**Table 1:** Textural properties and surface acidities of the prepared catalysts and pyridine adsorption with % yield of 7-hydroxy-4-methylcoumarin and 3,4-dihydropyrimidin-2(1*H*)-ones

Sample name	*S*_BET_ m^2^ g^−1^	Pore volume cm^3^ g^−1^	Initial potential (*E*_i_)	No. of acid sites in mequiv. g^−1^ (×10^19^)	B/L ratio	7-Hydroxy-4-methyl coumarin	3,4-Dihydropyrimidin-2(1*H*)-ones
Cr–Mg-MOF	1524	1.074	128.1	7.88	0.0548	0.0%	21.2%
10 wt% PMA/Cr–Mg-MOF	1352	0.975	218.4	8.18	—	25.7%	54.4%
25 wt% PMA/Cr–Mg-MOF	1108	0.723	337.7	9.19	0.6721	38.1%	75.4%
50 wt% PMA/Cr–Mg-MOF	957	0.662	399.1	9.49	0.9475	48.1%	81.3%
75 wt% PMA/Cr–Mg-MOF	745	0.487	496.1	13.98	1.4981	68.7%	96.1%
90 wt% PMA/Cr–Mg-MOF	512	0.331	424.6	13.22	1.157	61.2%	84.1%

**Fig. 6:**
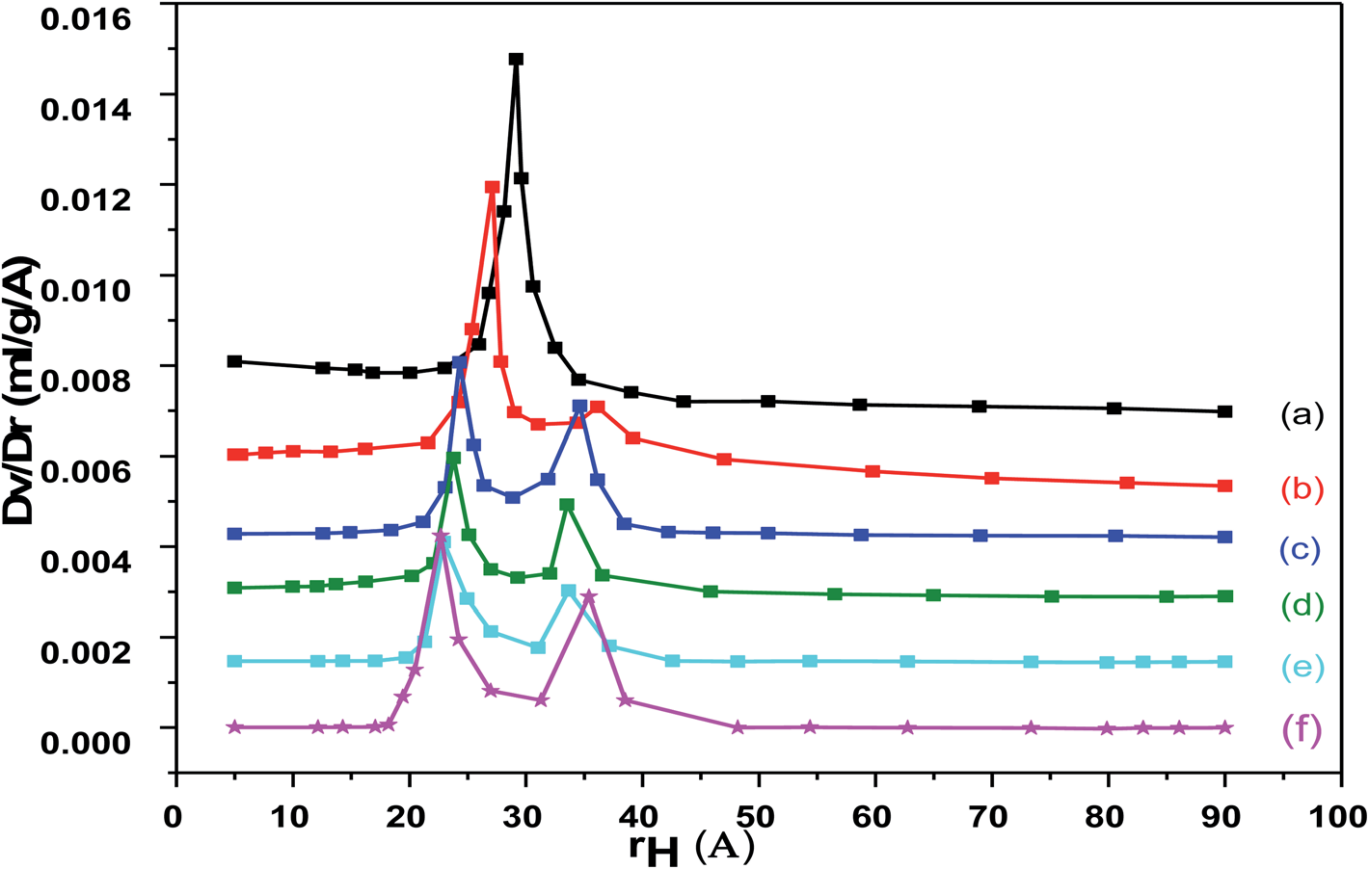
Pore volume distribution for (a) mixed Cr–Mg-MOF; (b) 10 wt% PMA/Cr–Mg-MOF; (c) 25 wt% PMA/Cr–Mg-MOF; (d) 50 wt% PMA/Cr–Mg-MOF; (e) 75 wt% PMA/Cr–Mg-MOF; (f) 90 wt% PMA/Cr–Mg-MOF.

The pore size distribution of pure and loaded Cr–Mg-MOFs shows a unique peak centered at about 28 Å diameters as shown in Fig. 6. It is evident that the pore volume and the specific surface area of the loaded sample are much lower compared to that of the pure Cr–Mg-MOFs. This is consistent with previous results shown in Table 1, the reduction in the pore volume and surface area after loading could be due to the fact that the PMA is deposited inside the mesochannels and is well dispersed on the surface of metal organic frameworks.

### 3.5 Acidity measurements

#### 3.5.1 Non-aqueous titration

Acidity measurements of Cr–Mg-MOFs and 10, 25, 50, 75 and 90 wt% of PMA loaded on mixed MOFs catalysts was measured by non-aqueous potentiometric titration which enables the determination of acidic strength and total number of acid sites existed over the catalysts. In order to understand the results, it is recommended that the maximum acidic strength of the surface sites can be measured from the initial electrode potential (*E*_i_) and the total number of acid sites of the catalysts calculated from the point where the plateau is touched as shown in the following relation:^31^
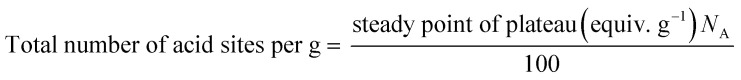
where, *N*_A_ is Avogadro's number.

**Fig. 7:**
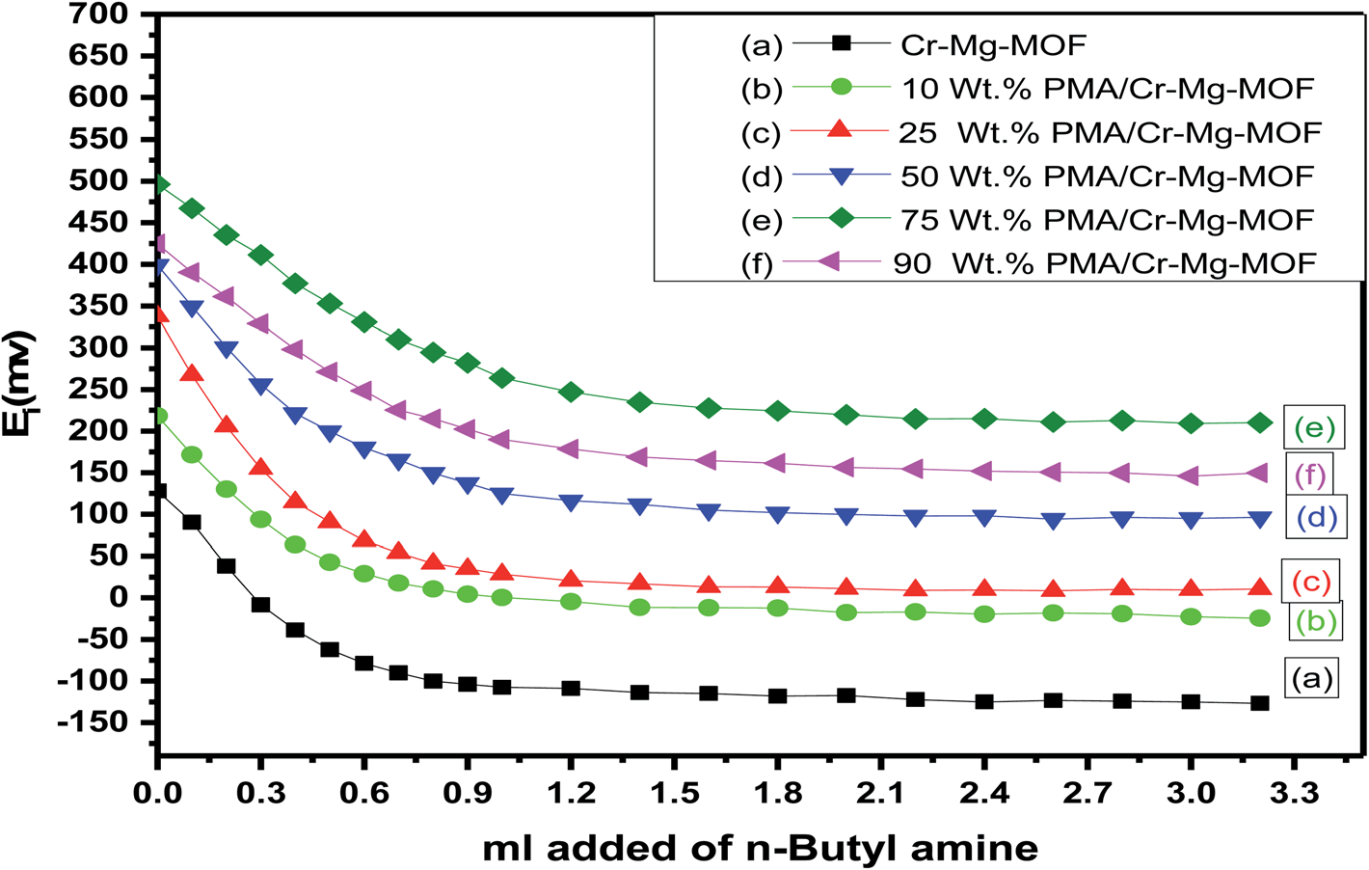
Potentiometric titration curves for (a) mixed Cr–Mg-MOF; (b) 10 wt% PMA/Cr–Mg-MOF; (c) 25 wt% PMA/Cr–Mg-MOF; (d) 50 wt% PMA/Cr–Mg-MOF; (e) 75 wt% PMA/Cr–Mg-MOF; (f) 90 wt% PMA/Cr–Mg-MOF.

**Fig. 8:**
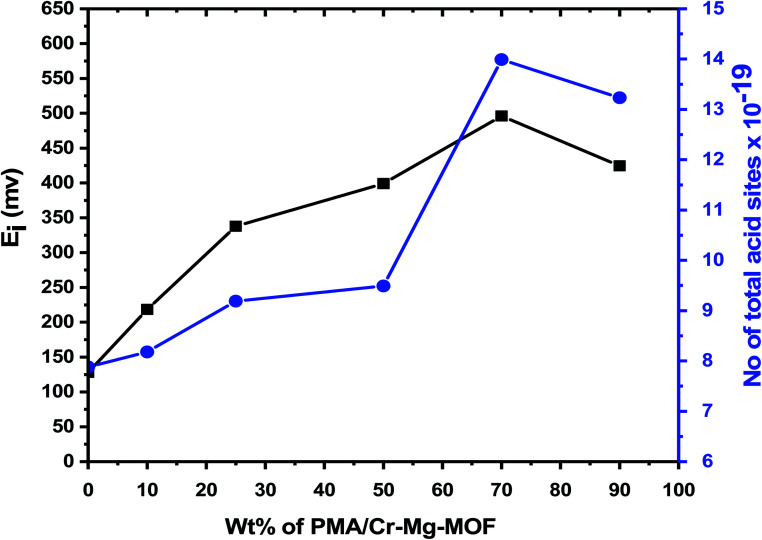
Total number of acid sites and initial potential *vs.* weight percent of PMA over Cr–Mg-MOF.

The titration curves of the as-synthesized catalysts were showed in Fig. 7 while the total number of acid sites and the acidic strength and *versus* the different weight percent of PMA were shown in Table 1 and Fig. 8. From the plot shown in Fig. 7 and 8, we find that the catalyst with 75% loading displays a very strong acid sites with initial potential equal (*E*_i_ = +496 mV) due to the good dispersion of PMA over mixed-MOFs. On the other hand, the total number of acid sites increased by increasing the PMA loading until 75 wt% and then decrease again which may be possibly due to the accumulation of PMA over the support as shown in Fig. 8 and Table 1.

#### 3.5.2 Pyridine adsorption

**Fig. 9:**
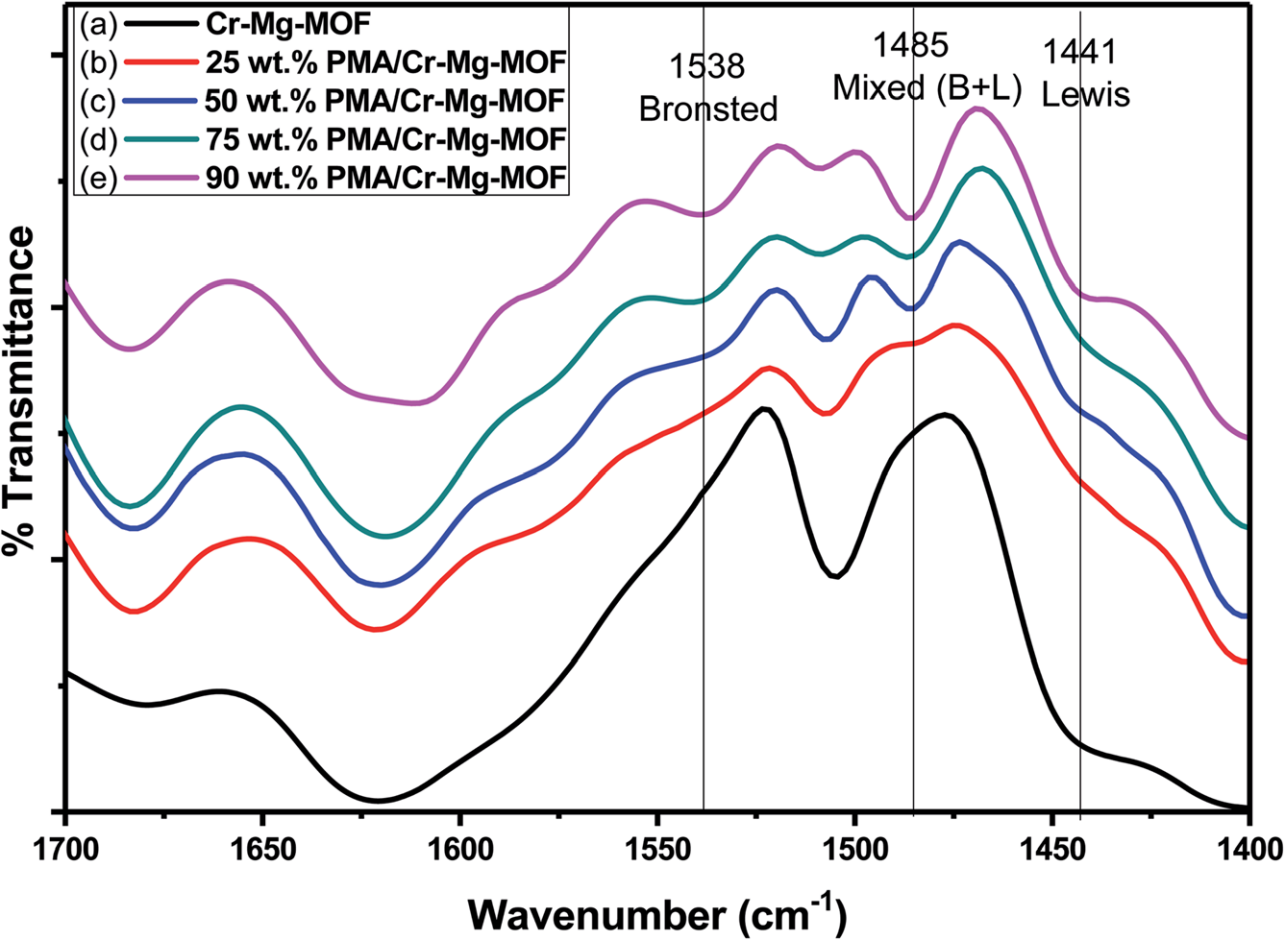
FT-IR spectra of chemisorbed pyridine on (a) mixed Cr–Mg-MOF; (b) 25 wt% PMA/Cr–Mg-MOF; (c) 50 wt% PMA/Cr–Mg-MOF; (d) 75 wt% PMA/Cr–Mg-MOF and (e) 90 wt% PMA/Cr–Mg-MOF.

The FTIR spectra of pyridine adsorbed on PMA/Cr–Mg-MOF contains 0, 25, 50, 75 and 90 wt% phosphomolybdic acid prepared by impregnation method then calcined at 150 °C is shown in Fig. 9. The pyridine adsorbed on Brønsted acid sites showing band at around 1538 cm^−1^ respectively. Moreover another band appears at 1441 cm^−1^ that corresponds to pyridine adsorbed on Lewis acid sites,^44^ this suggests that the supported catalysts had Brønsted acidity higher than the pure mixed MOFs. The bands at 1485 cm^−1^ correspond to mixed Brønsted and Lewis acid sites. According to the FT-IR spectra of chemisorbed pyridine, Cr–Mg-MOF showed both Brønsted and Lewis acid sites but the number of Lewis acid sites was higher compared with Brønsted acid sites. Mg played the major role in enhancing the Lewis acid sites while the Brønsted resulted from the linker (terephthalic acid).

The relative intensity of the band related to Brønsted acid sites increases gradually with the increase in PMA loading up to maximum at 75 wt% PMA/Cr–Mg-MOF, then the intensity decreases with further increase in PMA contents. The effect of PMA contents on the percentage of Brønsted to Lewis acid sites and the number of Brønsted acid sites are shown in Table 1 which indicates that, the percentage and the number of Brønsted acid sites increase with the increase in phosphomolybdic acid content up to 75 wt% PMA, then decrease again which may be due to the aggregation of the PMA crystals on the surface of PMA/Cr–Mg-MOF which results in decreasing the surface acidity.^45^

### 3.6 Catalytic activity measurement

#### 3.6.1 Synthesis of 7-hydroxy-4-methyl coumarin

##### 3.6.1.1 Effect of molar ratio

**Fig. 10:**
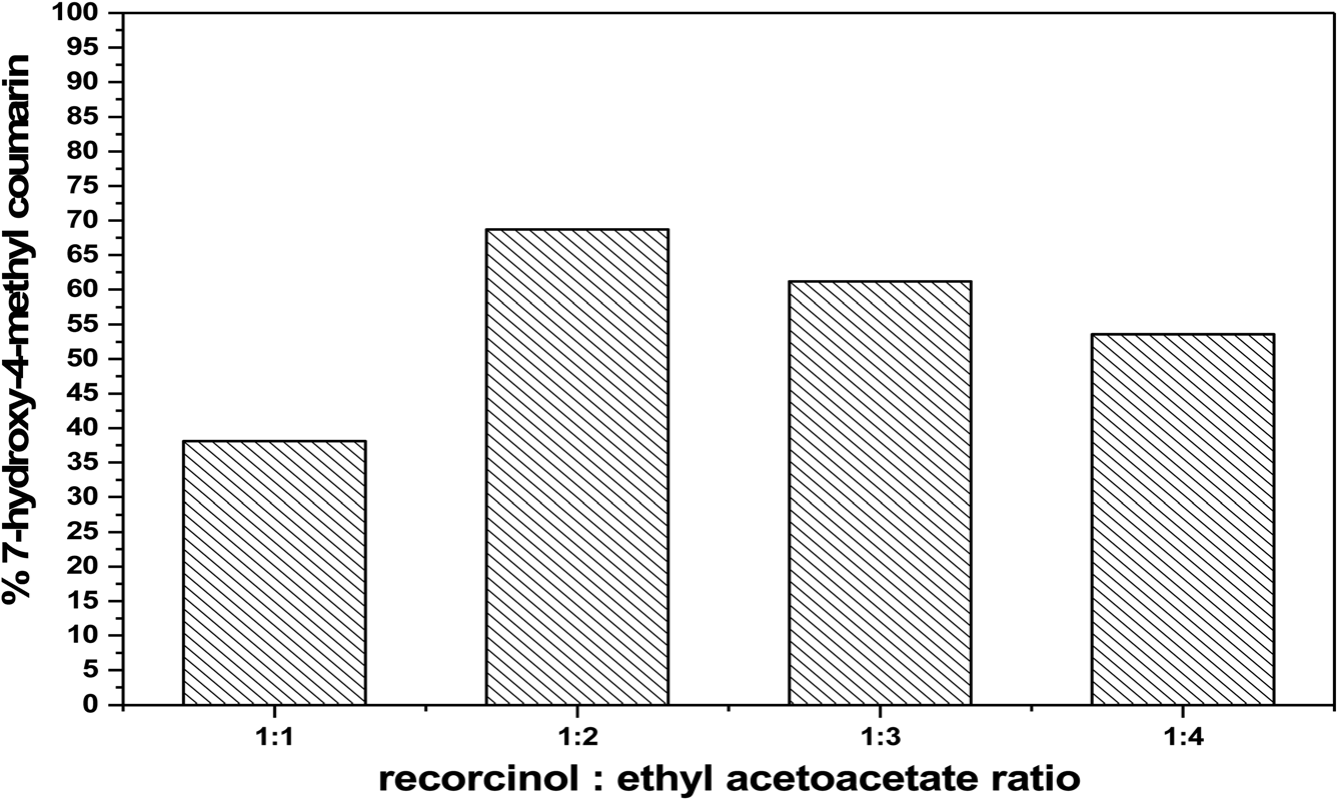
Effect of molar ratio of (ethyl acetoacetate : resorcinol) on the catalytic activity over 75 wt% PMA/Cr–Mg-MOF.

In a typical procedures, 2 mol ethylacetoacetate and 1 mol resorcinol (2 : 1) is the basic stoichiometry to yield 7-hydroxy-4-methyl coumarin. The ratio of ethyl acetoacetate to resorcinol was varied to examine the effect of molar ratio such as 1 : 1, 2 : 1, 3 : 1 and 4 : 1. The percentage yield was increased with increasing molar ratio up to 2 : 1 and then they promote the dehydration mechanism as shown in Fig. 10. One can propose that the promotion of removing the water molecules decreases, once the pores of the catalyst get blocked by the product precipitation.^46^

##### 3.6.1.2 Effect of reaction time, reaction temperature and catalyst weight

The effect of the reaction time, reaction temperature and the catalyst weight on the percentage yield of 7-hydroxy-4-methyl coumarin were examined over 0.05 gram of 75% PMA/Cr–Mg-MOF at 120 °C and a molar ratio 2 : 1 (ethyl acetoacetate : resorcinol). The reaction was trailed at different time intervals (monitored by TLC) up to three hours as shown in Table 2. The results displayed that product yield raises with rising the reaction time from 15 min to 120 minutes, and no important raise in the product yield was observed after 120 minutes. While, the effect of reaction temperature on the product yield of 7-hydroxy-4-methyl coumarin were examined. The temperature was varied from 80° to 140 °C. The results displayed that, the product yield increases with increasing the temperature to 120 °C and no significant increase in the percentage yield over 120 °C was observed as shown in Table 2.

**Table 2:** Optimization of reaction between resorcinol and ethyl acetoacetate in synthesis of 7-hydroxy-4-methylcoumarin over 75 wt% PMA/Cr–Mg-MOF

Entry	Amount of catalyst (g)	Temperature (°C)	Time (min)	Yield (%)
1	0.05	120	15	Trace amount
2	0.05	120	30	27.1%
3	0.05	120	60	38.1%
4	0.05	120	90	51.2%
5	0.05	120	120	68.7%
6	0.05	120	150	69.1%
7	0.05	120	180	70.2%
8	0.05	80	120	41.4%
9	0.05	100	120	51.7%
10	0.05	140	120	69.1%
11	0.02	120	120	48.1%
12	0.1	120	120	69.4%

On another hand, the percentage yield of the desired coumarin were investigated over different 75% PMA/Cr–Mg-MOF at different catalyst weight (0.02, 0.05 and 0.1 g) at 120 °C for 120 minutes. The results exhibited that the yield percentage increases from 48.1 to 68.7 then to 69.4 with increasing the catalyst weight from 0.02 into 0.05 into 0.1, respectively as displayed in Table 2. This indicate that the higher yield was attained at 0.05 g of catalyst and there was no important raise in the percentage yield after 0.05 gram of the catalyst. Therefore, according to the above results, the optimum temperature, time and catalyst weight for the synthesis of 7-hydroxy-4-methyl coumarin were 120 °C, 120 minutes and 0.05 gram, respectively.

##### 3.6.1.3 Effect of weight percentage of PMA

**Fig. 11:**
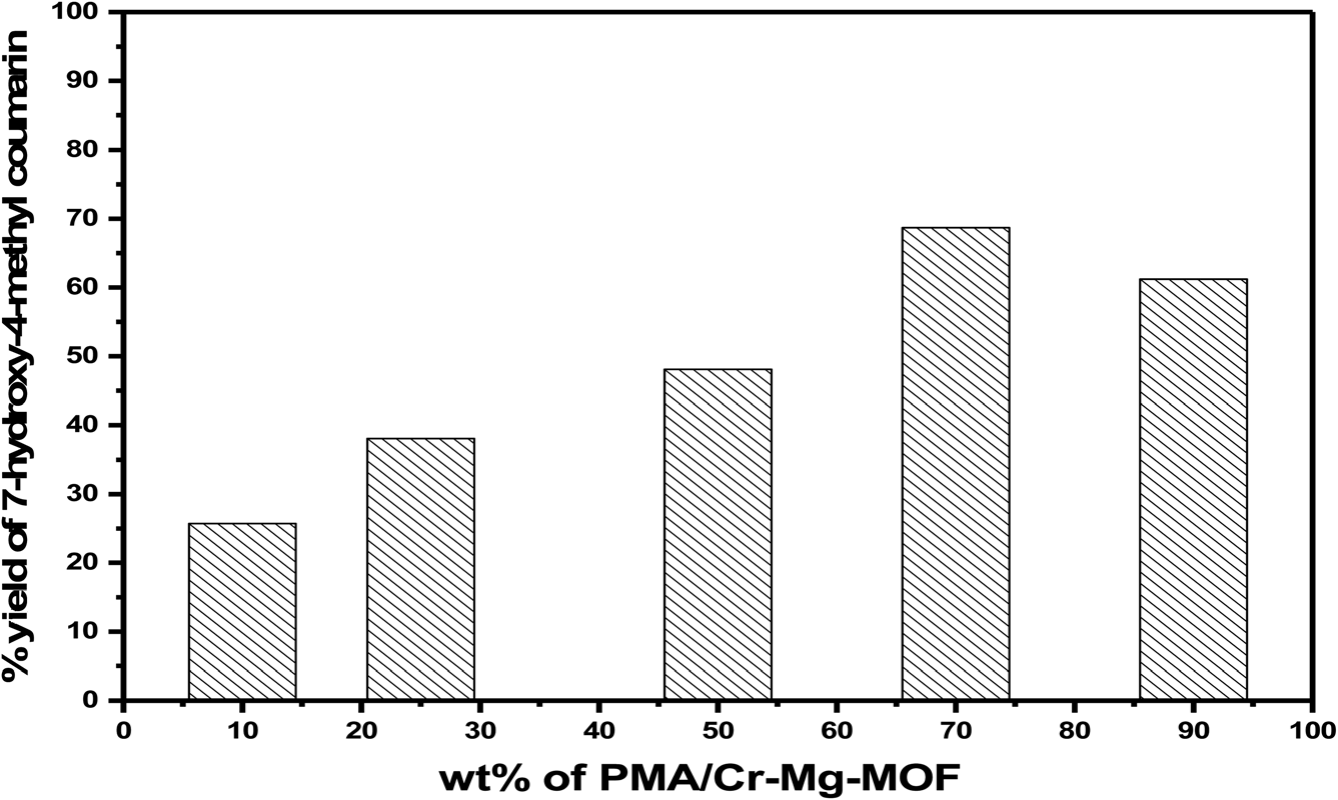
The relationship between PMA content and percentage yield of 7-hydroxy-4-methylcoumarin.

The catalytic activities of as-synthesized catalysts (*x* wt% PMA/Cr–Mg-MOF) were examined for synthesis of 7-hydroxy-4-methylcoumarin and the results displayed in Table 1. Pure Cr–Mg-MOF showed small catalytic performance in the reaction because of the weak acidic character and the synthesis of coumarin basically depends on Brønsted acid sites and many papers were proved the synthesis of coumarin depends only on the Brønsted acid sites^47^ as shown in non-aqueous potentiometric titration and adsorbed pyridine techniques. However, PMA/Cr–Mg-MOF with different weight percentage of PMA showed much higher activity. The product yield increases gradually with increasing the amount of PMA loaded on Cr–Mg-MOF until reach the maximum at 75 wt% PMA/Cr–Mg-MOF calcined at 150 °C then decreases again, which may be due to the decrease in the total number of acid sites and B/L ratio. Increasing the PMA above 75 wt% led to decreases % yield as displayed in Fig. 11. The reduction in activity may result due to the aggregation of the PMA crystals on the surface of PMA/Cr–Mg-MOF which responsible for the decreasing in both the surface acidity and catalytic activity.^45^ On the other hand, PMA alone was used as a catalyst and compared with the supported catalysts and the results showed that PMA/Cr–Mg-MOF had higher catalytic activity than PMA alone in which PMA catalyst produce a percentage yield equal 42%.

Comparison studies of the previously reported catalysts obtained for synthesis of 7-hydroxy-4-methylcoumarin with recent work (PMA loaded on Cr–Mg-MOFs) was displayed in Table 1S.† PMA/Cr–Mg-MOF acts as effective catalyst in the synthesis of the desired coumarin with respect to yields and products when compared with HKUST-1,^48^ Amberlyst-15,^49^ sulfamic acid@HKUST-1 (ref. 48) and W/ZrO_2_.^50^

##### 3.6.1.4 Reaction mechanism

**Scheme 1:**
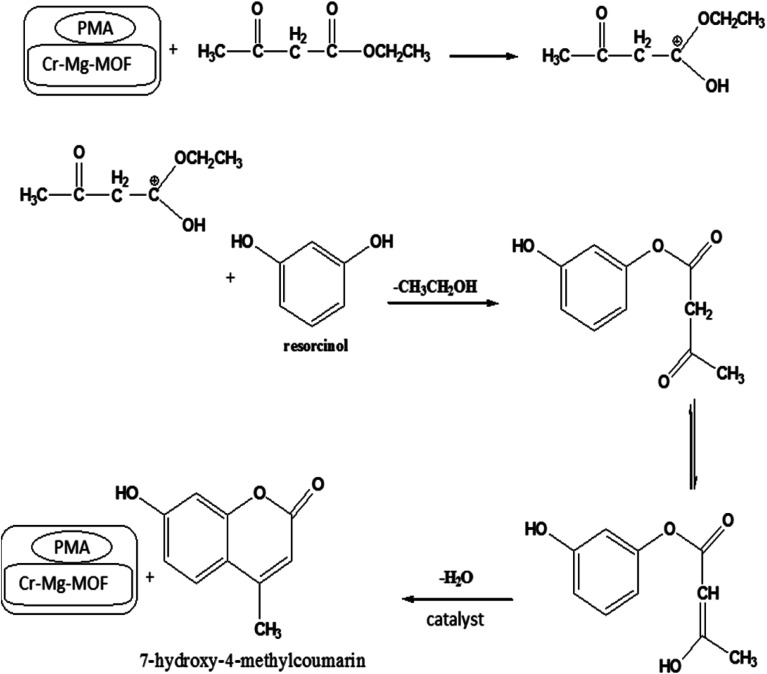
Reaction mechanism for the synthesis of 7-hydroxy-4-methyl coumarin catalyzed by PMA/Cr–Mg-MOF.

The mechanism of the synthesis of 7-hydroxy-4-methyl coumarin according to the above results can be suggested as follows: in the first step Brønsted acid protonates the carbonyl oxygen of ethyl acetoacetate by interaction of ethyl acetoacetate with catalyst which trigger the enhancement in electrophilic nature of carbonyl carbon. In the next step nucleophilic attack of the hydroxyl group of resorcinol on the carbonyl carbon of ethyl acetoacetate (electrophilic site) to give the intermediate complex which rapidly undergoes cyclization through intermolecular condensation yielding 7-hydroxy-4-methyl coumarin (Scheme 1).^51,52^

##### 3.6.1.5 Reusability of the catalyst

**Fig. 12:**
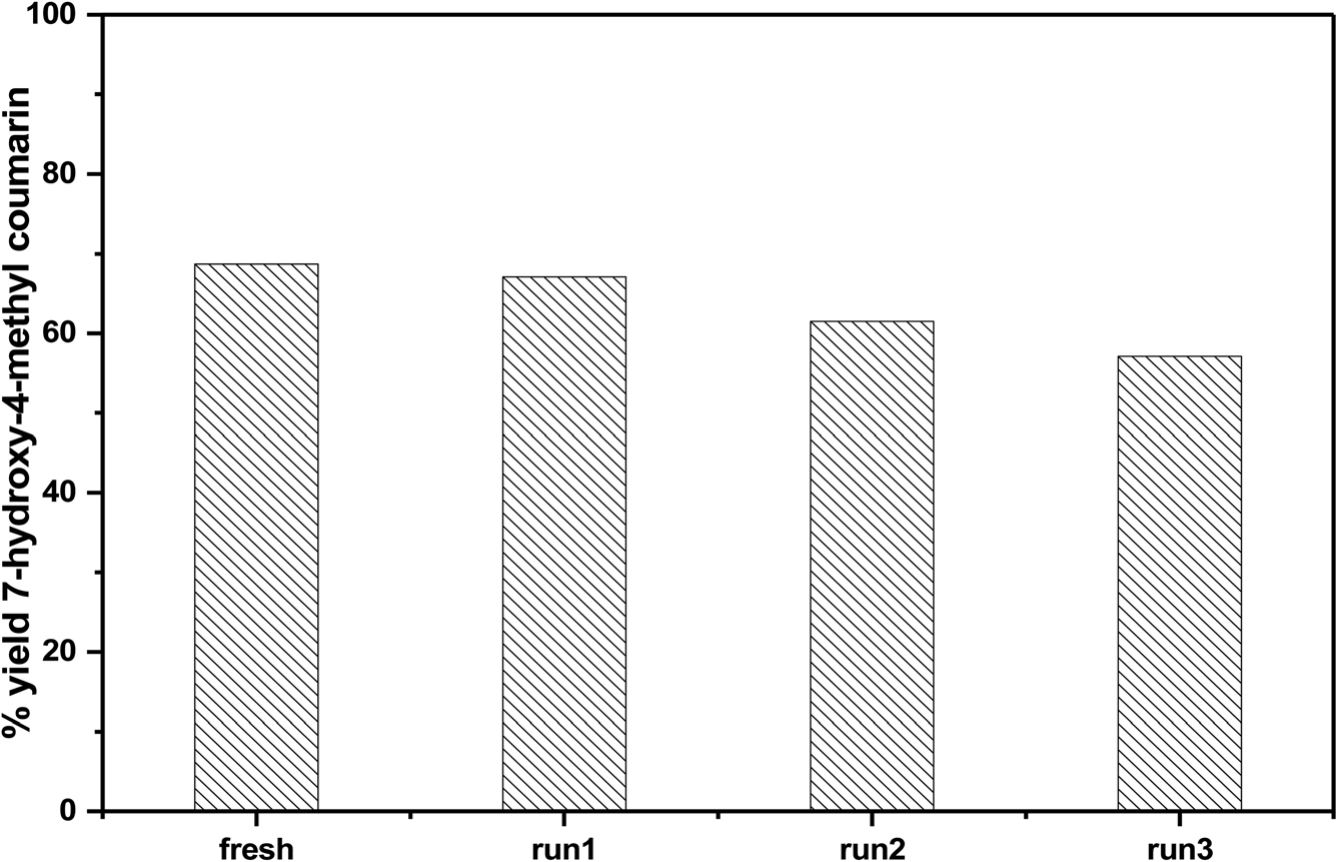
Reusability of 75 wt% PMA/Cr–Mg-MOF in the synthesis of 7-hydroxy-4-methylcoumarin.

In order to investigate the reusability of the as-synthesized catalysts, 75% PMA/Cr–Mg-MOF was tested four times for the synthesis of the desired coumarin under the same reaction conditions. The catalyst was separated from the reaction mixture by simple filtration process and washed with ethanol, then dried at 120 °C for 3–5 h to reuse in subsequent reaction. Fig. 12 shows that the catalyst displayed good reusability after four runs which indicate that the prepared catalysts could be reused different times without high loss in the catalytic activity. The stability of the catalyst was investigated *via* studying the stability of the characteristic IR bands before and after using of catalyst 4 times and there are small changes in the peak intensity indicating the stability of catalyst after reused 4 times.

#### 3.6.2 Synthesis of 3,4-dihydropyrimidinones

##### 3.6.2.1 Effect of molar ratio

**Fig. 13:**
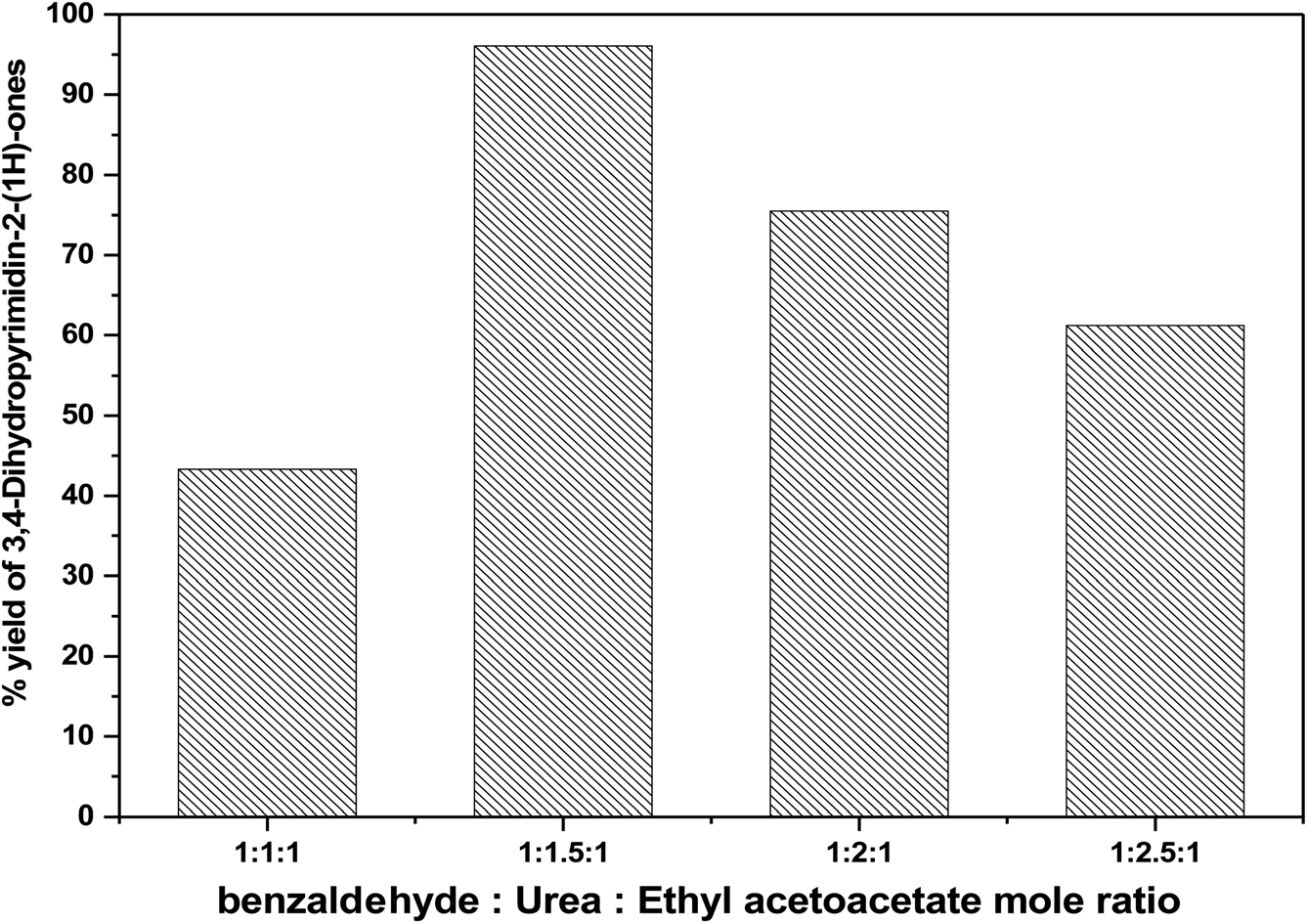
Effect of molar ratio of (benzaldehyde : urea : ethyl acetoacetate) on the catalytic activity over 75 wt% PMA/Cr–Mg-MOF.

Effect of molar ratio of on the formation of 3,4-dihydropyrimidinones over 75% PMA/Cr–Mg-MOF was tested by changing the ratio of benzaldehyde : urea : ethyl acetoacetate from 1 : 1 : 1 to 1 : 2.5 : 1 using 50 mg of catalyst at 100 °C for 60 min. It has been observed that the yield percentage increased from 43.32% to 96.1% with increasing the molar ratio 1 : 1.5 : 1. Extra increase in the molar ratio of reactants to 1 : 2 : 1 was attended to a significant decrease in the % yield to 87.3% as shown in Fig. 13. The reduction in catalytic activity might be clarified on the basis of the fact that the increases in the ethyl acetoacetate content hinder the reaction by blocking the active sites on the catalyst surface.^51,53,54^

##### 3.6.2.2 Effect of reaction time, reaction temperature and catalyst weight

Effect of the reaction time and temperature on the yield percentage of 3,4-dihydropyrimidinone were studied at 100 °C and a molar ratio 1 : 1.5 : 1 (benzaldehyde : urea : ethyl acetoacetate) using 0.05 g of 75% PMA/Cr–Mg-MOF. The reaction was tested at different time intervals (monitored by TLC) up to 2 h as shown in Table 3. The results showed that the percentage yield increased with increasing the reaction time from 15 min to 60 min, and no significant elevation in the percentage yield was observed after 60 min. While, the effect of reaction temperature on the percentage yield of 3,4-dihydropyrimidinone was examined at range from 80° to 120 °C. The % yield increased with rising the temperature up to 100 °C and no significant increase in the percentage yield above 100 °C was observed as shown in Table 3.

**Table 3:** Optimization of reaction between benzaldehyde : urea : ethyl acetoacetate in synthesis of 3,4-dihydropyrimidinones over 75 wt% PMA/Cr–Mg-MOF

Entry	Amount of catalyst (g)	Temperature (°C)	Time (min)	Yield (%)
1	0.05	100	15	Trace amount
2	0.05	100	30	61.2%
3	0.05	100	60	96.1%
4	0.05	100	90	96.7%
5	0.05	100	120	97.2%
6	0.05	80	60	60.1%
7	0.05	120	60	96.7%
8	0.02	100	60	60.1%
9	0.1	100	60	95.4%

On the other hand, the effect of catalyst weight on the percentage yield of 3,4-dihydropyrimidinones was examined over 75 wt% PMA/Cr–Mg-MOF at different weight catalyst (0.02, 0.05 and 0.1 g). The results showed that the yield percentage increased from 60.1 to 96.1 then to 96.7 with increasing the catalyst weight from 0.02 into 0.05 into 0.1, correspondingly. This indicates that the higher yield was achieved at 0.05 g of catalyst and there was no significant raise in the % yield after 0.05 gram of the catalyst as shown in Table 3. Therefore, according to the above results, the optimum temperature, time and catalyst weight for the synthesis of the desired 3,4-dihydropyrimidinone are 100 °C, 60 min and 0.05 gram, respectively.^55^

##### 3.6.2.3 Effect of weight percentage of PMA

**Fig. 14:**
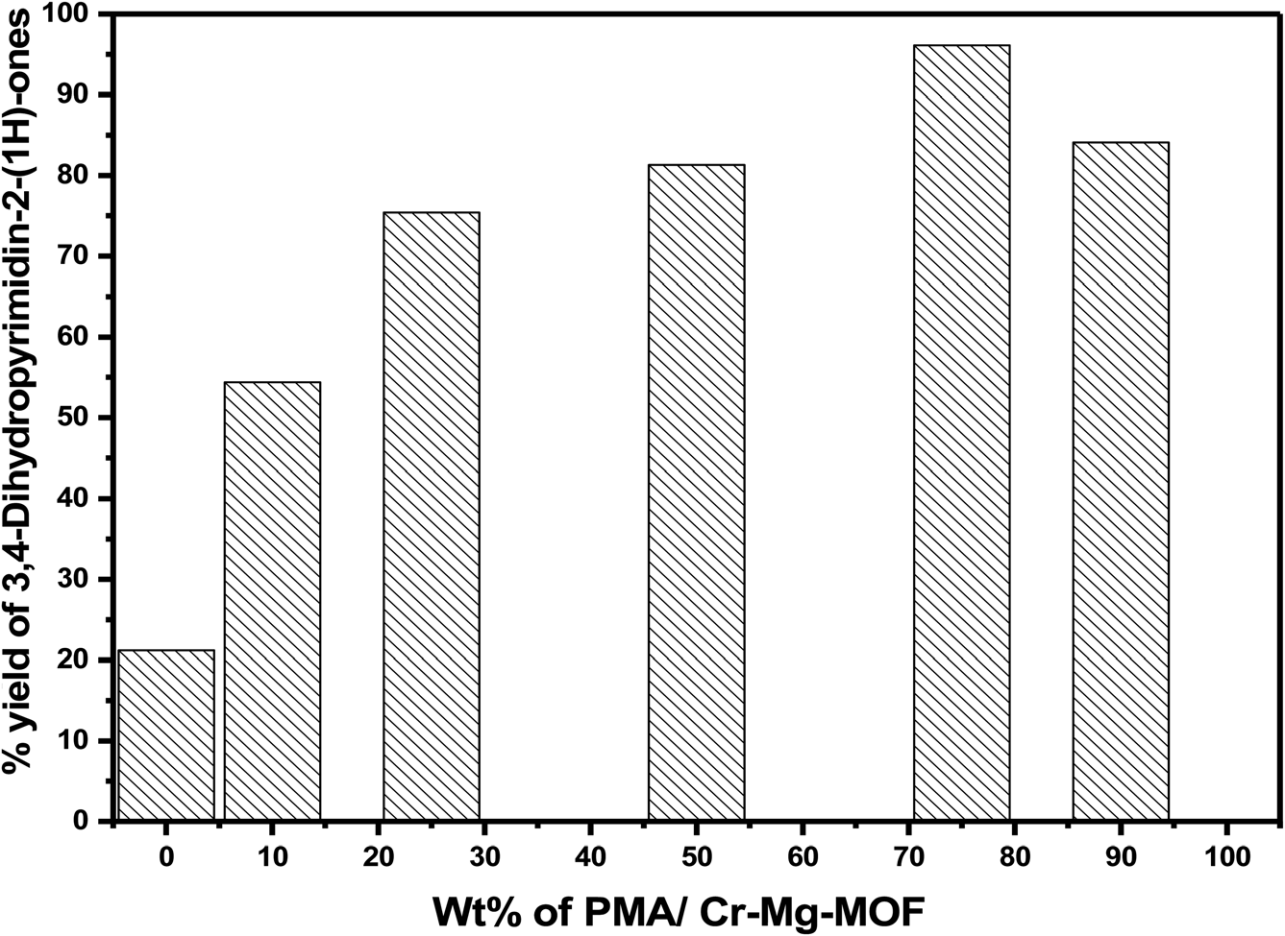
The relationship between PMA content and percentage yield of 3,4-dihydropyrimidinones.

The effect of PMA contents of PMA/Cr–Mg-MOF on the % yield of 3,4-dihydropyrimidinones was studied under the same reaction conditions. Mixed metal organic frameworks (Cr–Mg-MOF) showed small catalytic activity with percentage yield 22.1%, which may be due to the weak acidic active sites and this indicate that the synergetic effect between Mg and Cr metals enhances the acidic properties of the mixed MOF and increase the number of Lewis acid sites and produce a small yield of 3,4-dihydropyrimidinone. However, PMA/Cr–Mg-MOF with different contents of PMA show much higher activity. Among the catalysts with different PMA loadings, the 75 wt% PMA/Cr–Mg-MOF shows the highest activity with 96.1% yields of 3,4-dihydropyrimidinone. Increasing the PMA above 75 wt% led to decreases % yield as shown in Fig. 14. The decrease in activity may result due to the aggregation of the PMA crystals on the surface of PMA/Cr–Mg-MOF which results in decreasing both the surface acidity and catalytic activity.^31^ In comparison of the results obtained in this work using PMA loaded on Cr–Mg-MOFs with those achieved using HKUST-1,^48^ sulfamic acid@HKUST-1,^48^ cellulose sulfuric acid,^56^ silica sulfuric acid^56^ and AlCl_3_.^57^ The results show that PMA/Cr–Mg-MOF acts as effective catalyst in the synthesis of 3,4-dihydropyrimidinone with respect to yields and products as displayed in Table 2S.†

##### 3.6.2.4 Reusability of the catalyst

**Fig. 15:**
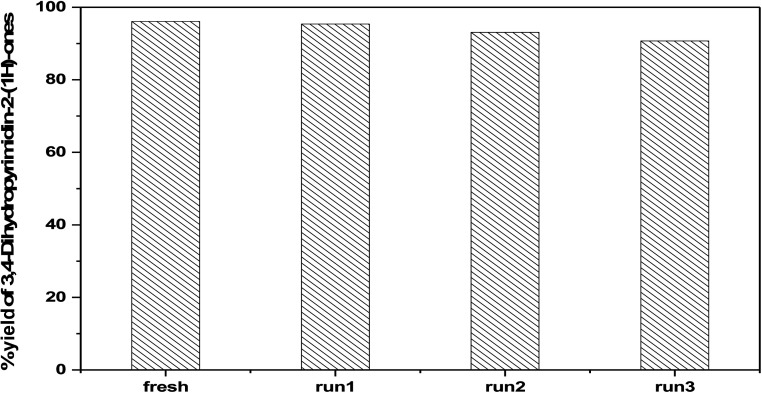
Reusability of 75 wt% PMA/Cr–Mg-MOF in the synthesis of 3,4-dihydropyrimidinones.

**Fig. 16:**
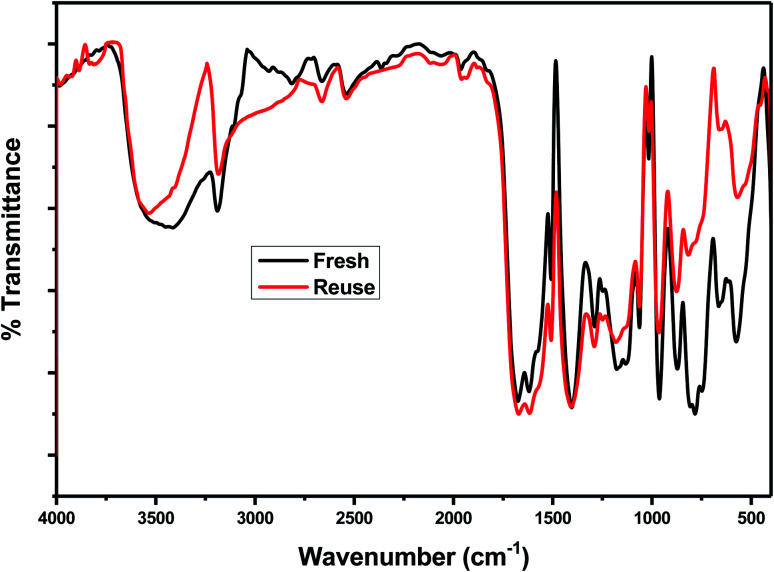
Effect of reuse on the structure of 75 wt% PMA/Cr–Mg-MOF catalyst.

The reusability of 75 wt% PMA/Cr–Mg-MOF was also checked with four times for the preparation of 3,4-dihydropyrimidinone under the same reaction conditions. The catalyst was separated from the reaction mixture by simple filtration and washed several times with ethanol, then dried at 120 °C for 3–5 h, Fig. 15 shows that the catalyst displayed good reusability after four runs which indicate that the prepared catalysts could be reused different times without high loss in the catalytic activity. Fig. 16 shows the change in the structure of 75% PMA/Cr–Mg-MOF catalyst before and after the reaction. Only the intensity of the bands were decreased and no change or new band appeared indicating the stability of catalyst after reused three times.

## 4. Conclusion

The results obtainable in this work provided a detailed description of the changes in the acidic features of series of phosphomolybdic acid loaded on mixed Cr–Mg-MOF. These catalysts were applied in synthesis of 7-hydroxy-4-methyl coumarin and 3,4-dihydropyrimidinones which give nearly the same yield as we use phosphomolybdic acid which means we can use these catalysts in green chemistry. The catalytic activity is related to the availability of Brønsted acid sites originated from PMA. The optimum conditions in synthesis of 7-hydroxy-4-methyl coumarin have been found that, the molar ratio of resorcinol : ethyl acetoacetate is 1 : 2 at 120 °C and reaction time is two hours. While, the optimum temperature, time and catalyst weight for the synthesis of the desired 3,4-dihydropyrimidinone are 100 °C, 60 min and 0.05 g, respectively. In the synthesis of 3,4-dihydropyrimidinone both Brønsted and Lewis acid sites can be catalyzing the reaction. So, Mg as a Lewis acidic sites played important role in catalyzing the reaction in parallel with Brønsted acid sites while the synthesis of 7-hydroxy-4-methyl coumarin is basically depends on the Brønsted acid sites and many papers were proved the synthesis of coumarin depends only on the Brønsted acid sites. But in case the synthesis of dihydropyrimidinone both Brønsted and Lewis acid sites can be catalyzing the reaction. Also, the addition of Mg together with Cr enhanced the acid strength and surface acidity due to the synergistic effect between two metal species. Also, the results illustrated that the % yield of 7-hydroxy-4-methyl coumarin and 3,4-dihydropyrimidinone are related to the availability of Brønsted acid sites originated from the Keggin structure of PMA anions which in turn depends of the PMA content.

## Conflicts of interest

There are no conflicts to declare.

## Supplementary Material

RA-010-D0RA03591B-s001
