# Correction: The role of PMA in enhancing the surface acidity and catalytic activity of a bimetallic Cr–Mg-MOF and its applications for synthesis of coumarin and dihydropyrimidinone derivatives

**DOI:** 10.1039/d4ra90020k

**Published:** 2024-03-07

**Authors:** Reda S. Salama, Shawky M. Hassan, Awad I. Ahmed, W. S. Abo El-Yazeed, Mohammed A. Mannaa

**Affiliations:** a Basic Science Department, Faculty of Engineering, Delta University for Science and Technology Gamasa Egypt reda.salama@deltauniv.edu.eg dr.reda.salama@gmail.com +201061391656; b Chemistry Department, Faculty of Science, Mansoura University Mansoura Egypt; c Chemistry Department, College of Sciences and Humanities in Al-Kharj, PrinceSattam Bin Abdulaziz University Al-Kharj 11942 Saudi Arabia; d Chemistry Department, Faculty of Science, Amran University Yemen mnnaam@yahoo.com +967714152023

## Abstract

Correction for ‘The role of PMA in enhancing the surface acidity and catalytic activity of a bimetallic Cr–Mg-MOF and its applications for synthesis of coumarin and dihydropyrimidinone derivatives’ by Reda S. Salama *et al.*, *RSC Adv.*, 2020, **10**, 21115–21128, https://doi.org/10.1039/D0RA03591B.

The authors regret that an incorrect version of Fig. 4 was included in the original article. The correct version of Fig. 4 is presented below.

**Fig. 4:**
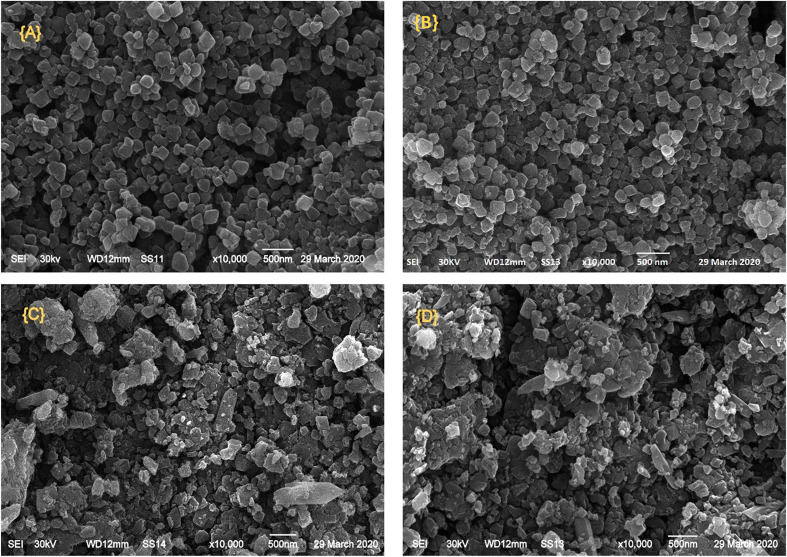
SEM images of (A) mixed Cr–Mg-MOF; (B) 25 wt% PMA/Cr–Mg-MOF; (C) 50 wt% PMA/Cr–Mg-MOF and (D) 90 wt% PMA/Cr–Mg-MOF.

The Royal Society of Chemistry apologises for these errors and any consequent inconvenience to authors and readers.

## Supplementary Material

