# Supplementary material for: The role of PMA in enhancing the surface acidity and catalytic activity of a bimetallic Cr–Mg-MOF and its applications for synthesis of coumarin and dihydropyrimidinone derivatives
Source: RSC Adv. 2020 Jun 3;10(36):21115–28. doi: 10.1039/d0ra03591b (PMC9054398; doi:10.1039/d0ra03591b)
Supplement: RA-010-D0RA03591B-s001 [file RA-010-D0RA03591B-s001.pdf]

Electronic Supplementary Material (ESI) for RSC Advances.
This journal is © The Royal Society of Chemistry 2020

The role of PMA in enhancing the surface acidity and catalytic activity of bimetallic Cr-Mg-MOF and its applications for synthesis of coumarin and dihydropyrimidinone derivatives

Reda S. Salama [a,*], Shawky M. Hassan [b], Awad I. Ahmed [b], W. S. Abo El-Yazeed [b, c], and Mohammed A. Mannaa [d,*]

[a] Basic Science Department, Faculty of Engineering, Delta University for Science and Technology, Gamasa, Egypt.

[b] Chemistry Department, Faculty of Science, Mansoura University, Mansoura, Egypt.

[c] Chemistry Department, College of Sciences and Humanities in Al-Kharj, Prince Sattam Bin Abdulaziz University, Al-Kharj 11942, Saudi Arabia

[d] Chemistry Department, Faculty of Science, Amran University, Yemen.

* Corresponding author at: Basic Science Department, Faculty of Engineering, Delta University for Science and Technology, Gamasa, Egypt, Tel.: 00201061391656.

* Corresponding author at: Chemistry Department, Faculty of Science, Amran University, Yemen. Tel.: 00967714152023.

E-mail address: reda.salama@deltauniv.edu.eg, dr.reda.salama@gmail.com (R. S. Salama), mnnaam@yahoo.com (M.A.Mannaa).

Table 1S†: Comparison study for synthesis of 7-hydroxy-4-methylcoumarin in presence of Cr-Mg-MOF and wt. % PMA/ Cr-Mg-MOF with other catalysts reported in the literature

Entry	Catalyst	Conditions	Yield (%)	Ref.
1	Without catalyst	Free solvent, Reflux, 120° C, 6hr	Nil	Recent work
	Cr-Mg-MOF	Free solvent, Reflux, 120° C, 2hr	Traces	Recent work
2	75 wt.% PMA/ Cr-Mg-MOF	Free solvent, Reflux, 120° C, 2hr	68.7 %	Recent work
3	HKUST-1	Free solvent, Reflux, 120° C, 2hr	2.0 %	[48]
4	Sulfamic acid@ HKUST-1	Free solvent, Reflux, 120° C, 2hr	59.0%	[48]
5	MIL-101 (Cr)	Free solvent, Reflux, 120° C, 2hr	Traces	[31]
6	Amberlyst-15	Free solvent, 150° C, 2hr	72.0%	[49]
7	W/ZrO_2	Toluene,150° C, 6hr	80.0%	[50]

Table 2S†: Comparison study for synthesis of 7-hydroxy-4-methylcoumarin in presence of Cr-Mg-MOF and wt. % PMA/ Cr-Mg-MOF with other catalysts reported in the literature

Entry	Catalyst	Conditions	Yield (%)	Ref.
1	Without catalyst	Free solvent, Reflux, 100° C, 6hr	Traces	Recent work
	Cr-Mg-MOF	Free solvent, Reflux, 100° C, 1.5hr	21.2 %	Recent work
2	75 wt.% PMA/ Cr-Mg-MOF	Free solvent, Reflux, 100° C, 1.5hr	96.1 %	Recent work
3	HKUST-1	Free solvent, Reflux, 80° C, 2hr	35.0 %	[48]
4	Sulfamic acid@ HKUST-1	Free solvent, Reflux, 80° C, 2hr	89.0%	[48]
5	cellulose sulfuric acid	CH_2Cl_2	68.0 %	[56]
6	silica sulfuric acid	Solvent, 100° C	91.0 %	[56]
7	$AlCl_3$	Solvent free, 8–10hr	40.0 %	[57]
